# Patient Information and Consent for Care in the Intensive Care Unit

**DOI:** 10.3390/healthcare11050707

**Published:** 2023-02-27

**Authors:** Jean-Philippe Rigaud, Fiona Ecarnot, Jean-Pierre Quenot

**Affiliations:** 1 Department of Intensive Care, Centre Hospitalier, 76202 Dieppe, France; 2 Espace de Réflexion Éthique de Normandie, University Hospital Caen, 14000 Caen, France; 3 Department of Cardiology, University Hospital Besançon, 25000 Besançon, France; 4 EA3920, University of Franche-Comté, 25000 Besançon, France; 5 Department of Intensive Care, Burgundy University Hospital, 21079 Dijon, France; 6 Lipness Team, INSERM Research Center LNC-UMR1231 and LabEx LipSTIC, University of Burgundy, 21078 Dijon, France; 7 INSERM CIC 1432, Clinical Epidemiology, University of Burgundy, 21078 Dijon, France

**Keywords:** intensive care, ethics, information, consent

## Abstract

In this paper, we review the ethical issues involved in providing information to, and obtaining consent (for treatment and/or research) from patients in the intensive care unit. We first review the ethical obligations of the physician in treating patients, who are by definition, vulnerable, and often unable to assert their autonomy during situations of critical illness. Providing clear and transparent information to the patient about treatment options or research opportunities is an ethical and, in some cases, legal obligation for the physicians, but may be rendered difficult, not to say impossible in the intensive care unit by the patient’s health state. In this context, we review the specificities of intensive care with respect to information and consent. We discuss who the right contact person is in the ICU setting, with possible choices including a surrogate decision maker, or a member of the family, in the absence of an officially designated surrogate. We further review the specific considerations relating to the family of critically ill patients, and the amount and type of information that may be given to them without breaching the tenets of medical confidentiality. Finally, we discuss the specific cases of consent to research, and patients who refuse care.

## 1. Introduction

In the field of medicine, ethical reflection is based on the four universal principles of biomedical ethics described by Beauchamp and Childress, namely beneficence, non-maleficence, autonomy and justice [1]. Acting in a manner that brings about a benefit, or at least does not result in harm, while respecting the autonomy of the patient, and providing equitable treatment for all, are the basic ethical prima facie obligations of any medical action.

Treating a patient, and doing what is right for them, is the daily duty of the physician, and the reverse side of the coin implies that the physician also should not bring about harm, by inflicting unnecessary physical or mental suffering. Respect for the patient is fundamental, and informing a sick patient about their disease, their treatment options, and their likely prognosis is the behavioural translation of that respect. The patients should be involved in the making of any decisions that concern them, because they have a unique perspective of their illness and how it affects them [2,3]. In this regard, patients are free to give due consideration to their situation and decide for themselves what is best for them, and they must be allowed to express their wishes regarding possible diagnostic tests or therapeutic approaches proposed by the physician.

The patient’s possibility to exercise their autonomy is intricately linked to the information that they receive from the physician, and to the expression of consent by the patient. Indeed, in order for a patient to properly weigh up his/her options and make the appropriate choice after giving all the options due consideration, without being unduly influenced, and in order to consent to the healthcare pathway that is being proposed, the patient has to have received clear, understandable, complete and accurate information about his/her condition. The quality of the information provided by the physician, and how it is understood by the patient, underpins the validity of the patient’s consent or refusal [4].

In medicine, the physician’s obligation to inform the patient carries potentially weighty consequences in terms of responsibility, if the duty is not carried out appropriately, since the patient’s right to participate in decisions pertaining to their health is established as a right, as is the right to receive good quality information. This is a reflection of changes in society, whereby the patient nowadays takes a more active role in their health, and patients’ rights are now enshrined in French legislation, in the various laws and amendments relating to patients’ rights and the quality of the healthcare system. The legislation emphasizes the need for full and accurate disclosure of information to the patient, so that they may express their wishes and take an active part in their healthcare [5].

However, exercising this right can be difficult for many patients. It is therefore incumbent on the physician to accompany the patient and guide them through this process. There are numerous clinical situations where the principle of autonomy can be difficult to respect, notably when the patient is not in a position to receive information, when the patient has difficulty understanding due to extreme mental, physical or social suffering, or when the patient is faced with a decision that carries weighty consequences [6]. Beyond the legal obligation, the physician also has a moral duty to protect and help their patient, who represents the epitome of the vulnerable person to whom we owe a duty of care and assistance. Indeed, the French code of medical ethics specifies that the physician has a duty to provide clear and accurate information to the patient, but the information must also be appropriate to the patient’s state and circumstances [7]. This leaves some leeway to adapt the information in the patient’s best interests, according to the clinical context, the patient’s personality or mental state [4]. In such situations, with the goal of beneficence, and without drifting into paternalism (the attitude whereby the physician decides for the patient without taking the patient’s opinion into account), the physician must guide, accompany, advise and support the patient in the decision-making process. This should guarantee that every patient has the opportunity to make decisions concerning their health, together with their healthcare professional, and taking account of the information and advice that was provided. Further, the physician must then respect the patient’s choice, after having informed the patient of the consequences of that choice. If the patient’s choice to refuse or discontinue care could put their life at risk, then the physician should make every effort to convince the patient to accept essential care [5].

French legislation also stipulates that patients should be informed about any complementary examinations, for the same reasons. Again, the code of public health stipulates that every patient has the right to be informed about their state of health, including the different investigations, treatments or preventive interventions being proposed, their utility, whether they are urgent or not, their possible consequences, any foreseeable, frequent or serious risks associated with them, the alternative solutions, and possible consequences of refusal. Here again, the physician is obliged to inform the patient, or someone representing the patient. Indeed, the law stipulates that no medical intervention or treatment can be initiated without the free and informed consent, and this consent may be withdrawn at any time. Furthermore, if the patient is unable to express their wishes, no intervention or investigation may be performed (unless it is urgent) unless a surrogate, family member or other representative has been consulted first.

When a patient is unable to receive, assimilate and/or understand information relating to their state of health from the physician, a surrogate or “reference person” may be designated to act on their behalf. This possibility was first instituted in France in 2002, and the law states that any adult (aged > 18 years) may designate a surrogate (who may be a relative, friend, or treating physician) to be consulted in case the patient is unable to express their wishes and/or receive the necessary information to make their own decisions.

The role of the surrogate in decision making was reinforced in subsequent versions of the laws relating to end-of-life issues, in 2005 and 2016 [8,9]. If we consider that the family knows what the patients’ wishes are, and that the family is beneficently disposed towards the patient, then it follows that the family would likely tend to act in the patient’s best interests. However, experience shows that this is not always the case, as families may have undue influence over the patient. Similarly, it has been argued that patients should be considered within the broader context of their familial group, allowing social space for families to be involved in decision making for their members [10,11]. Additionally, even for ICU physicians, there are also a number of competing interests that may result in decisions that are not necessarily in the best interests of an individual patient [12]. Therefore, the point of view of the family or surrogate must of course be taken into account, but in an advisory capacity only. The family’s decision should not be final if the physician believes it is contrary to the patient’s best interests or expressed desires. Furthermore, in the context of French legislation, patients have the right to designate an official surrogate, but the ICU physician is not obliged to adhere to this distinction.

As a corollary of information, the patient’s consent (or refusal) is a key component of the doctor–patient relationship. The individual’s right to freedom from interference in their health is a basic human right [13]. Physicians should be mindful that respecting this basic right is one of the pillars of the relationship of trust that exists between doctor and patient. Failing to respect this right can have deleterious, and potentially irreversible consequences on the quality of the doctor–patient relationship, as well as on the patient’s attitude towards the healthcare system in general [14]. An offer of medical intervention is the fruit of a balanced reflection on the risks of disease versus the benefits and potential disadvantages or adverse effects of treatment or research participation. All the arguments that weigh in the decision to propose a particular medical option should be shared with the patient, so that the information provided may enable the patient to choose what they believe to be the best of course of action. Providing detailed information about the background to an offer of medical intervention also makes it possible to explain the why and the how of the management process, so that the patient may understand, share any comments or doubts, then consent or refuse, and perhaps ask for assistance (from family, surrogate, other health professionals etc.), and if necessary, take all the necessary organizational dispositions, as appropriate [15]. Providing clear and accurate information helps to involve the patient in establishing their therapeutic goals of care together with the family and healthcare professionals, in a climate of shared decision making. This helps to ensure care that is aligned as closely as possible with the patient’s wishes and desires [16,17]. Nevertheless, respecting the patient’s autonomy should not blend into moral indifference towards the patient, nor should the physician seek to hide behind the principle of autonomy. While the patient is required to provide information to the patient about their diagnosis, prognosis, exams and treatments, they must also seek to identify what the patient wants to know, and is capable of hearing. To this end, they must be humane, notably by modulating the content of the information appropriately. The physician must respect the patient’s autonomy, but should not veer into total indifference vis-à-vis the patient; that would be highly reprehensible [18,19].

It should be noted that while it is the patient’s right to be informed (via a surrogate, where applicable), this does not necessarily go hand-in-hand with a request for consent in every situation. Indeed, when a withholding or withdrawal of life-support therapies is being considered, and if the patient is mentally incompetent or otherwise unable to express themselves, the decision is made by the medical team, on condition that the collegial decision-making procedure is respected, and that appropriate information about that decision is given to the family and/or surrogate [4,5,20].

## 2. Specificities of Intensive Care

In routine medical practice, the most commonly encountered situation is that of a patient who is conscious and capable of receiving relevant information about diagnosis and management, integrating that information, understanding its implications, consenting to care and complying with the proposed therapy or exams. Ideally, the patient’s representative or surrogate would be an integral part of this process.

Despite the existence of a legal framework, difficulties arise in situations of uncertainty, such as unconscious patients, persons with dementia, emergencies, psychiatric cases and patients with neurological disorders. All of these situations are frequent among patients admitted to the intensive care unit (ICU), and several of them may co-exist in a single patient. Admission to the ICU is a situation where it is only rarely possible for the patient to exercise their right to autonomy. In the vast majority of cases, at admission to the ICU, the patient is unconscious, under sedation, or their life is in the balance, and they are therefore quite unable to express their wishes or make decisions. In addition, the rapid decisions about urgent treatment initiation that such situations often require are not compatible with the time-consuming process of trying to find out what the patient’s wishes are.

Consequently, in such situations, the information is often provided to the family, or to the surrogate if one is designated, and to the patient’s general practitioner or other treating physicians. Despite generally being perfectly willing to provide all the relevant information, the intensivist is often prevented from doing so, because the admission may be an emergency or unexpected, or there may be no officially designated surrogate, and sometimes even no family members at all present at the time of admission. Frequently, at admission or during the ICU stay, the family are incapable of assimilating and understanding medical information, because of the anxiety and worry caused by the serious, not to say life-threatening illness that has suddenly beset their loved-one. Most of these aspects are beyond the direct control of the intensivist, and may not change in any substantial way during the patient’s stay in the ICU.

## 3. Who Is the Right Contact Person in the ICU?

### 3.1. A Surrogate Decision Maker

It is important to remember that a surrogate decision maker only exists if the patient him/herself has designated one. Neither the caregiving staff nor the family can designate an official surrogate if the patient has not done so. Therefore, if there is an officially designated surrogate, then medical information can be given to this person, and this person only. The physicians can carry out their job within the framework laid down in the legislation, and there are no limitations on the form or content of medical information that can be given, except if the patient has expressly opposed the transmission of certain information [5]. By designating an official surrogate, the patient establishes the type and extent of information that can be transmitted. However, this means that the physician caring for the patient has to accept that the designated surrogate is the “right” person, and the only person capable of receiving medical information, and of relaying and respecting the patient’s wishes. This situation is relatively straightforward when an official surrogate for the patient has been designated prior to admission. However, what should you do when there is no officially designated surrogate, a situation that is encountered almost daily in routine ICU practice? Or when an official surrogate has been designated, but does not have the patient’s best interests at heart, or the best interests of the family? In addition, how should one deal with the patient’s relatives, who are naturally worried and eager for information? It can happen that patients do not choose the most appropriate person as their surrogate, especially as regards the sharing and communicating of medical information. In a study about the information provided to families when further tests are required for a patient in the ICU [20], it was shown that most physicians almost systematically refer to “the family” or “the relatives” in a general way, and not specifically to “the surrogate”. In reality, while the legal provisions for an official surrogate exist in France, very few people actually have an officially designated surrogate, and therefore, the presence of “a surrogate” is quite rare in French ICUs. The reasons for this are manifold: emergency admissions for acute and unpredictable situations, failure to designate a surrogate in advance, as well as lack of awareness among healthcare professionals and the general public about the possibility to designate a surrogate. It is not our intention to call into question the principle of surrogate designation simply because it is difficult to apply in the context of critical care. While the confines of the 2002 law about patients’ rights and end-of-life issues are strict, it is almost certainly possible to take into account the specificities of ICU patients. Even while respecting the principle of autonomy, the physician still has a moral duty to protect patients [4,19], and it is therefore legitimate that this duty extends to protecting the family as well [21]. We will therefore discuss below the hypothesis that beyond the principle of autonomy, there is a need for a principle of beneficence towards the patient and their family.

### 3.2. Who to Inform When There Is No Surrogate?

It is logical to assume that the people who are at the patient’s bedside in the ICU are the family and close relatives (or people whom the patient would consider as such). These persons surely already possess a great deal of information about the patient’s prior health status, notably the pre-hospital course, the patient’s previous state of health at home, etc. Sometimes, the person(s) present at the bedside were also there when the doctor or the emergency services came to treat and transfer the patient. So, in general, they do know quite a lot about the patient and can be a precious source of information when the patient is admitted to the ICU. Given that the majority of people understand that admission to the ICU is synonymous with a very serious situation, it is also logical to assume that the persons present are already aware, to a certain extent, of the patient’s state, before they ever meet with the ICU physician.

When no official surrogate has been designated, which is the case in the vast majority of ICU admissions, it is useful to identify a “reference person” among the relatives who can act as a surrogate for the patient. This reference person must meet the caregivers’ expectations [21]. The physician should be able to identify, among all the relatives present, which of them is the best suited to act as surrogate, the most benevolent, perhaps the closest to the patient, and in any case, the most capable of receiving and understanding relevant medical information. The choice is far from being easy. Close relatives, especially the patient’s spouse, are usually the most available, and often the most capable of understanding the medical information and reporting the patient’s wishes [22]. However, the sudden hospitalization of their loved-one may cause them acute psychological distress, and prevent adequate comprehension or cloud their judgement. They may even require specific support themselves to manage their distress [18,23,24]. Indeed, it has been shown that those who struggle most in the role of surrogate decision maker are those who lacked prior experience as a surrogate and those who had no prior discussions with the patient about treatment preferences [25]. Accordingly, choosing a surrogate from among the relatives should not put that surrogate in difficulty, either personally, or vis-à-vis the other members of the family. It is also not acceptable to exclude any members of the entourage or family from the communication, once a surrogate has been chosen from among the relatives. On the contrary, it is equally impossible for the ICU physician to provide information to all the relatives, every day [18,20,21,26]. The involvement of families in decision making for critically ill loved ones is a vast and complex subject that has been widely discussed in the literature. Our group has previously reported on the challenges of obtaining consent in the ICU setting [27], while Azoulay et al. have extensively reported on the challenges of involving families in the care of, and decision making for critically ill patients in the ICU [23,28,29]. In these studies, it was shown that participating in decision making for critically ill loved ones increases the family’s satisfaction, but also that a substantial number of families do not wish to be involved in decision-making processes. Indeed, making decisions against a background of the psychological stress caused by the sudden hospitalization of a loved-one can cause significant distress and be an unwelcome burden for some. Therefore, any reluctance on the part of the family to participate in decision making must be heeded by the ICU physicians.

To cater for these complicated situations, it could be of interest to propose that a key contact person be chosen among the family members using a collegial decision-making process. The family, general practitioner, ICU staff and physicians could meet to designate the person to serve as the surrogate, according to a process that remains to be determined, but which could be modelled on the “family conference”, for example. Such a procedure would guarantee the legitimacy of the surrogate, avoid problems with the other family members, and perhaps also avoid having surrogates who struggle with their new responsibility [21]. There could even be two or more surrogates designated to share the responsibility. This type of procedure would not interfere in any way with the official designation of a surrogate by the patient while still in good health, but would be nonetheless well suited to the specific challenges of the ICU context. Similarly, as discussed below, an exception to the rule of medical secrecy, limited to the duration of the ICU stay, could also be envisaged, justified by the principle of beneficence towards the patient’s family [21].

### 3.3. Taking the Family into Consideration

The family’s capacity to understand medical information is an important aspect to take into account. In our previous work [21], we reported that the family’s poor capacity for comprehension was cited by physicians as a factor in their choice of a suitable surrogate. The physicians seem to engage in more intense communication in such cases, but may also tend to limit the amount or type of information imparted, to avoid putting the family into difficulty, either out of beneficence towards them, or because the physician feels that the family will simply not be able to understand or assimilate the information. Conversely, it is noteworthy that compared to their younger counterparts, older and/or more experienced physicians are significantly less prone to limit information, but rather adapt their language appropriately [20].

In the ICU, the vulnerability of the patient’s family evolves in parallel to that of the patient. The family are eager for a word, a gesture, or a titbit of information that might relieve their stress even a little. The family is in a situation of complete dependence on their loved-one’s outcome, and on the medical and caregiving teams, who hold the key to the diagnosis, treatment and information. A lack of communication can give rise to post-traumatic stress disorder, with resultant repercussions on daily functioning, and the possible need for psychological assistance [30,31].

In the ICU setting, numerous factors contribute to almost instantaneous and constant transmission of medical information to the families, such as: the constant presence of medical staff, the availability of the caregivers, the intensity of the care delivered, the large range of complementary exams performed, the increasingly long visiting hours, the wide spectrum of variability in the clinical state of patients, and the uncertainty surrounding prognosis. An oversight or an inaccurate piece of information can usually be corrected in time, before—aided by the family’s anxiety—a state of incomprehension or loss of trust in the medical team sets in.

Absent the possibility to have a meaningful relationship with the patient, the caregivers in the ICU have a very particular relationship with the family. They must at all times show benevolence towards the family, and assist them as they deal with the difficult situation of their loved-one’s hospitalization. In this way, there is a shift from “what is right for the patient” towards “what is right for the family”. Building and cultivating this relationship of trust, notably via pertinent communication, certainly contributes to creating the climate of trust that is essential between the caregivers and the family. In addition, this also promotes high quality management of the patient, and most likely also increases the family’s overall satisfaction [25,32].

Apart from being a key component of the quality of care, provision of adequate information meets a strong need expressed by the families, whose anxiety and “need to know” are understandable. In addition to the legal aspects of providing information in the ICU setting, it is important to take account of the relatives’ need for information. They are constantly waiting for some news about the status of a loved-one with whom they cannot communicate, and the information given to them must therefore be transparent, relevant and adapted to their level of comprehension. When a family is worried and desirous of information about the health status of a loved-one who is unable to express themselves, then providing that information is undoubtedly an act of benevolence.

There is one final issue that can arise with regard to information to families of ICU patients, and that is, what would the patient have wanted? Would the patient have agreed for specific information about his/her prognosis to be delivered indiscriminately to the family members? Is there any possibility that the information about the patient’s prognosis, given to the family by the physician, could affect the family, or their personal lives, or their future projects or future commitment to the patient? When the prognosis is uncertain, poor or abysmal, telling the family as much will inevitably cause them distress. This must be considered during discussions with the family, and it behoves the ICU physician to be particularly prudent and empathetic when addressing this point in conversations with the family.

## 4. What Information Should Be Given to the Family of a Patient Hospitalized in the ICU

Currently, there is no legal obligation to provide exhaustive information to the families of ICU patients. Insofar as, in practice, families are kept informed, it is legitimate to raise the question of the form and content of the information to be given: how far can or should the ICU physicians go? In addition, what leeway do they have with regard to the respect for medical confidentiality [20,21]?

### 4.1. The Type of Information

The question of the type of information to be given to the families of ICU patients is at the interface between medical confidentiality and the family’s capacity to understand. Medical confidentiality is intended to protect the patient, and preserve their image, their future and their past. It is easy to understand that the information given to the family of an unconscious ICU patient should not be detrimental to the patient in any way. In this context, it would be acceptable to envisage a principle whereby the content and/or extent of information could be modulated according to personal, clinical, diagnostic and therapeutic data, but also according to the person receiving the information. It would be up to the physician to judge the quantity and quality of information that it is appropriate to deliver in each situation. In this regard, it should be remembered that, although we assume the family has the patient’s best interests at heart, they are nonetheless also bound by duties and obligations regarding the information they receive, notably confidentiality.

In the ICU setting, it is established that the type of information delivered to the patient’s family usually concerns the diagnosis, the expected course of disease and the key components of the treatment [18]. Estimates of the prognosis are more difficult, because the prognosis may often be hard to predict, especially in the early stages of the ICU stay. Therefore, information about prognosis should be cautious, and various communication strategies can be used to facilitate such conversations [33,34].

A second important element in the information to families relates to the need for additional investigations [20]. For the family, the fact that their loved-one is undergoing additional tests can reassure them as to the medical management. However, they may also be worried, and will inevitably be asking for the results of any additional investigations. It may even happen that the family expresses surprise that no such investigations are being performed, or that a patient as seriously ill as their loved-one should be in the ICU without undergoing additional investigations—as if the management of critically ill patients absolutely had to comprise a battery of complicated tests. Clinical judgement can pale in comparison to “indisputable” results from a blood test or CT scan. It can be hard to justify the “clinical judgement” approach when faced with questions from the family. It is reasonable for the family to believe that the results of additional tests might be important to counterbalance what the physician has told them, because they may perceive the physician’s discourse to be variable or inaccurate, despite the physician’s best efforts to be cautious and patient. Additional investigations, and their results, can appear to be more reliable, more concrete that just the doctor’s word. Indeed, it is commonplace, when non-urgent tests are being scheduled, such as a CT scan for persistent coma, for the family to consider it as a milestone in the patient’s pathway. For the physician, upcoming tests can provide an opportunity to inform, but also to stall for time, give perspective or materialize the stages of the patient’s clinical course.

Despite the increasing trend towards electronic health records in the ICU, one of the main sources of information remains the patient’s chart, a veritable treasure trove of information that brings together data about vital signs, treatment, biology and microbiology results, traceability of all additional tests and procedures, and key clinical events. The patient’s chart is almost universally available to the family in the ICU, and it is not unusual to see family members rush to check the chart first thing, as soon as they enter the patient’s room. It represents reliable and indisputable testimony to the events that occurred during their absence. It is also not unusual for the family to question the physicians about any changes or inconsistencies that they notice when reading the patient’s chart. We cannot emphasize enough how important it is to take the utmost care when filling in the patient’s chart, given how much it reflects (in the family’s eyes) the attention paid to the patient, and the quality of management.

### 4.2. Respecting Medical Confidentiality

The first and easiest approach to keeping medical information confidential is to give information to the official surrogate only. However, this policy has some shortcomings, as previously outlined. Another approach is the more pragmatic approach practiced in many ICUs, whereby a wider range of information is given, or to a wider circle of family members, justified by their right and need to know. This raises the question of the confidentiality of medical information in this context. It is evident that in view of current practices, absolute secrecy is increasingly difficult to uphold in the setting of the ICU. Indeed, the current legal framework was not conceived with the ICU setting in mind. It is impossible to ignore the high levels of distress suffered by the families of ICU patients, and they cannot be denied the empathy that is their due. Consequently, and inevitably, these considerations override the duty of medical confidentiality. If an individual wants information and has a right to that information (especially if they are related to someone hospitalized in the ICU), then information covered by the rule of medical confidentiality can be revealed with the sole aim of informing and reassuring the worried relatives. When seen in this light, transgressing the rule of secrecy could be seen as legitimate, and *in fine*, merely translates the reality of observed practices in the ICU, which may not follow the legal framework.

In summary, information is most often provided to family members out of necessity, in the absence of an officially designated surrogate, and out of empathy, due to their legitimate state of worry. The ICU physician is obliged to adapt his/her practices in order to meet the obligation to provide transparent and full information. This approach to providing information may lead to practices that fall outside the framework defined by current legislation, particularly as regards respecting medical confidentiality.

### 4.3. Information and Consent to Research

In clinical research involving human subjects [35], clear and honest information must be given to the potential participant, and the research may commence only after the patient has provided informed consent [13]. It should be underlined here that accepting to be enrolled in a clinical research project implies that one also accepts the limits of medical knowledge. It is easy to imagine the difficulties that this assent creates for patients and families, at a time when they are experiencing an unexpected and serious clinical situation, whose outcome remains uncertain. The information process cannot and must not be reduced to obtaining consent alone, if it is signed, written consent. It also cannot simply be limited to the content of the leaflets given to the patient, because such an approach is not appropriate for the clinical situation or the doctor–patient relationship [6]. Information about participating in research must be the starting point for a relationship of trust between the patient and the physician, which should be maintained throughout the duration of the research, and even beyond. Accordingly, sending the results of the research project to the patients may give them insights into their personal situation [15]. Here again, for the purposes of research in the ICU, the question arises of how decisions can be made by patients who are unable to communicate. In such situations, the patient’s surrogate decision making gives consent on the patient’s behalf, although often without knowing whether the patient would have wished to participate or not, and sometimes even in the knowledge that the patient probably would not have accepted to participate [36]. In this regard, contrary to the frequent distrust of experimental research, we should take into account the feeling families might have that management will be better if the patient is included in a clinical trial. In these conditions, it is easy to imagine that the family may not be totally serene when making these decisions. Therefore, even more than for medical care, the patient and family must be protected, and the physician cannot be absolved of his/her responsibility towards them, just because informed consent was obtained [15].

## 5. Refusal of Care

Respecting the patient’s autonomy is intricately linked to the principle of information. Providing information introduces the possibility for the patient to give consent, but also and equally, the possibility to refuse to consent. The patient’s autonomy is upheld by the surrogate or family when the patient cannot do so. So how should the ICU physician react when the surrogate or family refuses to consent to care or investigations that the medical team deems essential or necessary? Does the principle of informing the family mean that the physician should accept a flat refusal by the family on the patient’s behalf? Physicians should be mindful that seeking consent from the family opens the door to a possible refusal, which may be inopportune. Indeed, the families do not necessarily have the appropriate medical expertise to decide, and this can expose the patient to avoidable danger if an ill-advised refusal of care is taken into account. Furthermore, asking the family for consent may be in vain if they do not know what the patient would have wanted.

Physicians do not systematically take into account a refusal pronounced by the family in the patient’s best interests, even though they are presumed to be striving to uphold the patient’s bests interests. The principle of beneficence overrules the principle of autonomy in such cases. In routine practice, ICU staff are often confronted with ethical dilemmas where there may be conflict between two moral principles, notably between the principle of autonomy, and the principle of beneficence or non-maleficence [37]. The possible distress this conflict of values may cause the caregiving staff can be attenuated by including caregivers in the decision-making process [38]. However, the attitude where beneficence overrides autonomy could be considered by some to be paternalistic. Indeed, the balance between beneficence and autonomy is the foundation of the paternalistic model [39]. Even if patients have little, if any medical knowledge, they are still the best placed to make decisions for themselves, but to do so, they need to have access to the information that only the physician can provide [40]. The physician is no longer the only guarantor of what is best for the patient. Today, doctor–patient relationships have evolved towards an exchange between equals, in a context of shared decision making, whereby, in the ICU, decisions belong to the whole group, encompassing the patient, the nursing staff, the doctors and the families and/or surrogates.

We would like to underline that the type of situation being discussed here should not be confused with paternalism. Respecting the principle of autonomy is difficult when the patient is in a coma or sedated in the ICU. The fact that a physician may decide to ignore the family’s refusal of care or examinations that the physician deems indispensable, in the patient’s best interests, is a situation that falls outside the scope of what is covered by the definition of paternalism. We are dealing here with the case of patients who are incapable of receiving information or giving consent, and where the family’s decisions should not go against the patients’ best interests. We are not in a situation where the patient is fully capable of making a decision, but where the physician decides in (what they consider to be) the patient’s best interests, without taking the patient’s wishes and expressed preferences into account. That is, by definition, a paternalistic attitude, and it should not be confused with the specific situations that can arise in the ICU. In the same way, it is equally important to promote other attitudes, such as the duty to deliver care for the patient’s good, if that patient is unconscious. ICU physicians are accustomed to such situations, for example when there is a need for diagnostic surgery (an invasive procedure with inherent risks and a need for general anaesthetic), solely based on the physician’s medical opinion that it is in the patient’s best interests. We could also imagine this situation the other way round: failure to act in such a situation would be contrary to the patient’s best interests, and likely also an infringement of the respect for life, of the principle of beneficence, and of the principle of non-maleficence.

One key point to keep in mind in understanding behaviours in the situations discussed here is the potential reversibility of the incompetent state of ICU patients. Contrary to patients with dementia or irreversible neurological damage, the disease states encountered in the ICU, and which are responsible for the coma or need for sedation, will, in the majority of cases, have a favourable outcome for the patient, with complete neurological recovery. In view of their expertise and skills, ICU physicians are capable of identifying attitudes and decisions that might compromise a positive outcome for the patient. The extremely volatile and changeable nature of ICU outcomes argues for placing trust in the intensivist’s expertise as regards the level of therapeutic engagement. A family may refuse care that the intensivist deems indispensable, because they are anxious, worried, lack knowledge or maybe are in denial of the seriousness of the situation. A refusal expressed in such a context can be discounted if it is likely to compromise the patient’s outcome. Of course, it is necessary to hear a refusal expressed by the family, just as the physician would do if it was a conscious patient expressing the refusal, but at the same time, if that refusal is at odds with the collegial philosophy, then the medical team is not obliged to accept it. Indeed, it would not be benevolent towards the family to allow them to carry such a burden of responsibility. The physician, especially the ICU physician, is considered, in line with the principle of collegiality, as the guarantor of the patient’s bests interests at the end of life [8,9]. The same statute logically applies during curative therapy.

## 6. Conclusions: Information and Consent in the ICU—A Move Away from “Patient Alone” towards “Patient and Family”?

In the ICU setting, physicians make a concerted effort to inform the families of patients, not necessarily to obtain consent at all costs, but rather, out of benevolence towards the family. The process of providing information is complicated by the emergency context, the frequent absence of family members at admission or inability to contact them, and the need to respect medical confidentiality and/or the patient’s wishes. Communication can also be hampered by the lack of availability of the physicians, but also very often because there is no designated legal surrogate with whom the medical team can communicate. When a patient is unable to exercise their autonomy, should we systematically seek to designate a substitute or surrogate, who will be given free rein to exercise the patient’s autonomy? Indeed, French legislation gives patients the possibility to designate such a surrogate. However, the current legal framework does not adequately cover the actual conditions encountered in the ICU, notably the need of worried families to receive information about their loved-one’s state, and the physicians’ desire to show them appropriate empathy in such situations.

There is a need for a specific legal framework for delivering information in the ICU setting, whereby the intensivist would have more leeway with regard to respecting the patient’s autonomy, and would be able to give precedence to the principle of beneficence towards the patient AND the family. While the principle of autonomy is not to be ignored, it may be temporarily overridden by the principle of beneficence, in the form of increased attention to, and accompaniment of the family. This is especially true at a time when the patient is incapable of receiving information or making decisions, but when the responsibility of exercising the patient’s autonomy on his/her behalf may be particularly burdensome for a third person (even if they are a loved-one), regardless of whether they have been officially designated as surrogate or not. The principle of beneficence is founded on a moral duty to the weakest and most fragile among us—weak and fragile like our patients and their families.

## Data Availability

Not applicable: no new data were created.

## References

[1] Beauchamp T.L., Childress J.F. (2019). Principles of Biomedical Ethics.

[2] Perrotin D., Gold F., Choutet P., Burfin E. (1998). Ethique en réanimation. Reperes et Situations Éthiques en Médecine.

[3] Luce J.M. (1990). Ethical principles in critical care. JAMA.

[4] Moutel G. (2004). Le Consentement dans les Pratiques de Soins et de Recherche: Entre Idéalismes et Réalités Cliniques.

[5] (2011). Law 2002-303 Dated 4 March 2002 Regarding Patients’ Rights and Quality of Healthcare. http://www.legifrance.gouv.fr/affichTexte.do?cidTexte=JORFTEXT000000227015.

[6] Appelbaum P.S. (2007). Clinical practice. Assessment of patients’ competence to consent to treatment. N. Engl. J. Med..

[7] Conseil National de l’Ordre des Médecins Code of Medical Deontology. https://www.conseil-national.medecin.fr/code-deontologie.

[8] (2005). Law 2005-370 Dated 22 April 2005 Regarding Patients Rights and End-of-Life. http://www.legifrance.gouv.fr/affichTexte.do?cidTexte=JORFTEXT000000446240&categorieLien=id.

[9] (2016). Law 2016-87 Dated 2 February 2016 Introducing New Rights for Patients and Persons at the End-of-Life. https://www.legifrance.gouv.fr/affichTexte.do;jsessionid=46038F123BBBFC8DA2E8DE0EEE161860.tpdila19v_1?cidTexte=JORFTEXT000031970253&categorieLien=id.

[10] Ho A. (2008). Relational autonomy or undue pressure? Family’s role in medical decision-making. Scand. J. Caring. Sci..

[11] Cherry M.J. (2015). Re-Thinking the Role of the Family in Medical Decision-Making. J. Med. Philos.

[12] Turnbull A.E., Sahetya S.K., Biddison E.L.D., Hartog C.S., Rubenfeld G.D., Benoit D.D., Guidet B., Gerritsen R.T., Tonelli M.R., Curtis J.R. (2018). Competing and conflicting interests in the care of critically ill patients. Intensive Care Med..

[13] Lemaire F. (2007). The 60th anniversary of the Nuremberg doctor’s trial: Why a so long waiting to implement the Code?. Med. Sci..

[14] Pallocci M., Treglia M., Passalacqua P., Tittarelli R., Zanovello C., De Luca L., Caparrelli V., De Luna V., Cisterna A.M., Quintavalle G. (2023). Informed Consent: Legal Obligation or Cornerstone of the Care Relationship?. Int. J. Environ. Res. Public Health.

[15] Moutel G. (2013). Ethics of biomedical research: Questions about patient information. Med. Sci..

[16] Giammatteo J., Treglia M., Pallocci M., Petroni G., Cammarano A., Quintavalle G., Marsella L.T., Potenza S. (2020). LAW n.219/17: Reflecting on shared care plan. Clin. Ter..

[17] Quenot J.P., Ecarnot F., Meunier-Beillard N., Dargent A., Large A., Andreu P., Rigaud J.P. (2017). What are the ethical questions raised by the integration of intensive care into advance care planning?. Ann. Transl. Med..

[18] Azoulay E., Outin H., Pochard F., Saulnier F., Bion F. (2000). Information à la famille du patient en réanimation. Management en Réanimation—Evaluation, Organisation et Éthique.

[19] Jonquet O., Nicolas F., Rameix S., Offenstadt G. (2016). Information et consentement aux soins en réanimation. Réanimation.

[20] Rigaud J.P., Moutel G., Quesnel C., Eraldi J.P., Bougerol F., Pavon A., Quenot J.P. (2016). How patient families are provided with information during intensive care: A survey of practices. Anaesth Crit. Care Pain Med..

[21] Rigaud J.P., Hardy J.B., Meunier-Beillard N., Devilliers H., Ecarnot F., Quesnel C., Gelinotte S., Declercq P.L., Eraldi J.P., Bougerol F. (2016). The concept of a surrogate is ill adapted to intensive care: Criteria for recognizing a reference person. J. Crit. Care.

[22] Azoulay E., Pochard F., Chevret S., Adrie C., Bollaert P.E., Brun F., Dreyfuss D., Garrouste-Orgeas M., Goldgran-Toledano D., Jourdain M. (2003). Opinions about surrogate designation: A population survey in France. Crit. Care Med..

[23] Azoulay E., Chevret S., Leleu G., Pochard F., Barboteu M., Adrie C., Canoui P., Le Gall J.R., Schlemmer B. (2000). Half the families of intensive care unit patients experience inadequate communication with physicians. Crit. Care Med..

[24] Breu C., Dracup K. (1978). Helping the spouses of critically ill patients. Am. J. Nurs..

[25] Majesko A., Hong S.Y., Weissfeld L., White D.B. (2012). Identifying family members who may struggle in the role of surrogate decision maker. Crit. Care Med..

[26] Roupie E., Santin A., Boulme R., Wartel J.S., Lepage E., Lemaire F., Lejonc J.L., Montagne O. (2000). Patients’ preferences concerning medical information and surrogacy: Results of a prospective study in a French emergency department. Intensive Care Med..

[27] Ecarnot F., Quenot J.P., Besch G., Piton G. (2017). Ethical challenges involved in obtaining consent for research from patients hospitalized in the intensive care unit. Ann. Transl. Med..

[28] Azoulay E., Pochard F. (2003). Communication with family members of patients dying in the intensive care unit. Curr. Opin. Crit. Care.

[29] Azoulay E., Pochard F., Chevret S., Arich C., Brivet F., Brun F., Charles P.E., Desmettre T., Dubois D., Galliot R. (2003). Family participation in care to the critically ill: Opinions of families and staff. Intensive Care Med..

[30] Azoulay E., Pochard F., Kentish-Barnes N., Chevret S., Aboab J., Adrie C., Annane D., Bleichner G., Bollaert P.E., Darmon M. (2005). Risk of post-traumatic stress symptoms in family members of intensive care unit patients. Am. J. Respir. Crit. Care Med..

[31] Rigaud J.P., Quenot J.P., Bruyère R., Chahraoui K., Laurent A., Bioy A., Quenot J.P., Capellier G. (2015). Enjeux cliniques de la réanimation. Vulnérabilité Psychique et Clinique de L’extrême en Réanimation.

[32] Kon A.A., Davidson J.E., Morrison W., Danis M., White D.B., American College of Critical Care M., American Thoracic S. (2016). Shared Decision Making in ICUs: An American College of Critical Care Medicine and American Thoracic Society Policy Statement. Crit. Care Med..

[33] White D.B., Ernecoff N., Buddadhumaruk P., Hong S., Weissfeld L., Curtis J.R., Luce J.M., Lo B. (2016). Prevalence of and Factors Related to Discordance About Prognosis Between Physicians and Surrogate Decision Makers of Critically Ill Patients. JAMA.

[34] Burelli G., Berthelier C., Vanacker H., Descaillot L., Philippon-Jouve B., Fabre X., Kaaki M., Chakarian J.C., Domine A., Beuret P. (2018). Impact of a visual aid on discordance between physicians and family members about prognosis of critically ill patients. Anaesth. Crit. Care Pain Med..

[35] Law 2012-300 Dated 5 March 2012 Relating to Research Involving Human Subjects. https://www.legifrance.gouv.fr/loda/id/JORFTEXT000025441587/.

[36] Warren J.W., Sobal J., Tenney J.H., Hoopes J.M., Damron D., Levenson S., DeForge B.R., Muncie H.L. (1986). Informed consent by proxy. An issue in research with elderly patients. N. Engl. J. Med..

[37] Quenot J.P., Rigaud J.P., Prin S., Barbar S., Pavon A., Hamet M., Jacquiot N., Blettery B., Herve C., Charles P.E. (2012). Suffering among carers working in critical care can be reduced by an intensive communication strategy on end-of-life practices. Intensive Care Med..

[38] Quenot J.P., Rigaud J.P., Prin S., Barbar S., Pavon A., Hamet M., Jacquiot N., Blettery B., Herve C., Charles P.E. (2012). Impact of an intensive communication strategy on end-of-life practices in the intensive care unit. Intensive Care Med..

[39] Wolf M. (2015). Consentement, éthique et dogmes. Ethics Med. Public Health.

[40] Jaunait A. (2003). Comment peut-on être paternaliste? Confiance et consentement dans la relation médecin-patient. Raisons Polit..

